# Reduction of hydrocarbon pollutants by hyacinth plants ( *Eichhornia crassipes*)

**DOI:** 10.12688/f1000research.131846.2

**Published:** 2023-09-12

**Authors:** Syahril Nedi, Irwan Effendi, Afrizal Tanjung, Elizal Elizal

**Affiliations:** 1 Faculty of Fisheries and Marine, Universitas Riau, Pekanbaru, Riau, 28293, Indonesia

**Keywords:** Hydrocarbon pollutants, Hyacinths, Phytoremediation, TPH, Chlorophyll

## Abstract

**Background:** The application of phytoremediation by utilizing plants has been used to control oil pollution in waters. One of the plants that can act as a phytoremediator is the hyacinth because this plant can reduce various pollutants including petroleum hydrocarbons. This study aims to study the reduction ability of petroleum hydrocarbons at different concentrations including improving water quality.

**Methods:** This study consisted of one treatment (petroleum hydrocarbon) consisting of five factors with three replicates. The treatments consisted of 10 ppm (E1), 30 ppm (E2), 50 ppm (E3), 70 ppm (E4), 90 ppm (E5), and (E0) without aquatic plants as controls. The treatments were observed daily and measured from the first day (D-1), the seventh day (D-7), and the 14 ^th^ day (D-14). The water quality in each treatment was also measured, such as water temperature, pH, and dissolved oxygen.

**Results:** The results showed that the hyacinth plant was able to reduce hydrocarbon in terms of total petroleum hydrocarbon (TPH) by 79% while it was only between 17–27% naturally without the hyacinth. The reduction of TPH in the water was in line with the decrease of chlorophyll in the leaves of hyacinths, and it was followed by the increase of dissolved oxygen in the water media.

**Conclusions:** In conclusion, hyacinths can reduce petroleum hydrocarbons and they can improve the water quality as well.

## Introduction

Oil pollution is a common problem in coastal waters; such as oil slicks or spills. Most of the oil contains toxic hydrocarbon compounds that are vulnerable to habitats and biota. Common ways to encounter this pollution are through the oil boom, skimmer, dispersant, and bioremediation. ^1^^–^^7^ Bioremediation is a way to clean up contaminants in an environment by living organisms; for example, the use of bacteria and water plants.

Water hyacinth is one of plants that can be used as phytoremediator. This plant is easy to find in freshwater both in land and coastal area. Beside it grows faster, high adaption in nature. ^8^ This plant has ability to absorb pollutant with faster proliferation in a water. ^9^

Research on using hyacinth as a phytoremediator has been well documented ^10^^–^^12^ including to industrial waste. ^13^^,^^14^ Furthermore, this plant can also reduce about 99.5% of hexavalent chromium (Cr(VI)) in waste water, as well as to reduce TDS, BOD, and COD content. ^14^ Even though this plant can also be used as phytoremediator to heavy metal such as Hg and MeHg attaching on leafs of plants. ^15^

According to Priya and Selvan, water hyacinth can be used as a bioindicator of heavy metals in aquatic ecosystems. ^16^ In addition, water hyacinth as phytoremediators is cost-effective and eco-friendly and well-documented. ^17^^,^^18^ Based on this circumstance, the author is interested in testing the ability of the hyacinth plant to reduce hydrocarbon pollutants in saline water.

## Method

### Time and place

This research was conducted in June 2022 at the Lagio Laboratory, Pekanbaru. The test plant, hyacinth, was taken from the reservoir of Binawidya Campus, Faculty of Fisheries and Marine, Universitas Riau. Meanwhile, the hydrocarbon pollutants used come from Pertamina DEX Solar from Pekanbaru gas stations. The seawater was taken from the coastal waters of Dumai and composted fertilizer was also used for the test plants. The plants were grown in 20 L plastic washbasins.

### Research methods

This experiment used Completely Randomized Design (CRD) method with one factor and five levels of hydrocarbon concentrations treatments; three replications of each treatment. The treatments were: E1 = 10 mg/L, E2 = 30 mg/L, E3 = 50 mg/L, E4 = 70 mg/L, E5 = 90 mg/L and E0 = without plant. The E0 played as a control to know the natural evaporation and transpiration during observation. The reductions of the petroleum hydrocarbon in the treatments were measured in terms of total petroleum hydrocarbon (TPH).

### Procedure

*Acclimatization to salinity*

The container used was a black tub with a volume of 10 L of 15 units. The research was started by acclimatizing hyacinth plants to different salinities, namely 1, 3, 5, 7, and 9 ppt. In each container, 200 g of hyacinth was added and its growth was observed for seven days. Plant growth was seen based on changes in leaf color, root condition, and number of leaves. The acclimatization results showed that the test medium with a salinity of 3 ppt was suitable for plants to grow in coastal areas, some plants at salinity 5,7, and 9 died after being given salinity treatment.

*Petroleum hydrocarbon observations*

After determining the suitable salinity for the test plants, they were then planted in petroleum hydrocarbon pollutant solutions with different concentrations, namely 10, 30, 50, 70, and 90 mg/L, and without plants (control). The first step was a black tub with a volume of 20 L of 15 units filled with 10 L of water with a salinity of 3 ppt. Then 50 g of fertilizer was added to each container and 200 g of test plants were added. After that, petroleum hydrocarbons were added according to the concentration determined and observed for 14 days. ^19^^,^^20^ The observations were conducted from the first day (D-1), seventh day (D-7), and fourteenth day (D-14) by using ASTM 7066-04 (FTIR) method.

The parameters observed were total petroleum hydrocarbons, water quality (temperature, pH, DO, and salinity), and plant conditions (leaf color, root condition, and stem shape). To maintain the salinity in the test container, fresh water was added according to a predetermined volume limit, i.e. 10 L. Each container was marked, to determine the level of water whose volume is 10 L.

*Leaf chlorophyll measurement*

The measurement of chlorophyll was carried out with chlorophyll meter (KWF type). It was carried out by placing the hyacinth leaf in the sensor area of the equipment; the reading could be occurred between seconds. The Chlorophyll measurements of the treatments were conducted *in situ* between 10.00 AM–03.00 PM.

### Data analysis

Data on TPH concentration and chlorophyll were tabulated and analyzed using SPSS version 23. Data were analyzed using a *One Way ANOVA* with a confidence level of 95% and continued by a *Student Newman Keuls* (SNK) test if necessary.

## Result

### Total petroleum hydrocarbon concentration

The phytoremediators (hyacinths) could grow well in the tested media; with 3 ppt of saline water at 10–20 cm in height after acclimation. The content of hydrocarbon pollutants can naturally decrease, this also occurs in the treatment without the addition of plants; this is thought to be due to evaporation (E0). The concentrations of TPH in all treatments at the time of observations can be seen in Table 1.

**Table 1.** The concentrations and the reduction of total petroleum hydrocarbon (TPH) in each media of treatments on each observation day.

Treatment/TPH con. (mg/L)	TPH concentration (mg/L)				Total reduction (mg/L)
	D-7		D-14		
	Con.	Red.	Con.	Red.	
E1 (10)	3.70±0.20	6.30±0.02 ^a^	2.10±0.10	1.60±0.08 ^a^	7.90±0.10 ^a^
E2 (30)	17.33±0.76	12.67±0.76 ^b^	11.23±0.25	6.10±0.51 ^bc^	18.77±0.25 ^b^
E3 (50)	23.73±1.25	26.27±1.25 ^d^	19.07±0.95	4.66±0.30 ^b^	30.93±0.95 ^d^
E4 (70)	37.07±1.05	32.93±1.05 ^e^	29.27±1.25	7.80±0.20 ^c^	40.73±1.25 ^e^
E5 (90)	42.73±1.95	47.27±1.95 ^f^	33.73±0.25	9.00±1.70 ^d^	56.27±0.25 ^f^

Notes: E1 (10 mg/L); E2 (30 mg/L); E3 (50 mg/L); E4 (70 mg/L); E5 (90 mg/L); The superscript on the same line shows that there is an effect between treatments (P<0.05).

Table 1 showed that the TPH in all treatments reduced after the seventh day (D-7) and the 14 ^th^ day (D-14) of observations. The reductions were higher on the seventh day in comparison to the 14 ^th^ day of observation. These reductions ranged from 6.3–47.27 mg/L and from 1.6–9 mg/L consecutively. However, the total reductions ranged from 7.9–56.27 after the 14 ^th^ day of observations. This means that the longer the observation time the higher the TPH reductions. The affectivity of the plant to reduce TPH showed the same pattern due to the time constraint of observations.

In addition, there was a significant effect of hydrocarbon concentrations on the plant’s ability to reduce TPH, based on ANOVA analysis that p<0.05.

This shows that water hyacinth is able to absorb hydrocarbons up to a concentration of 90 mg/L.

**Figure 1.**
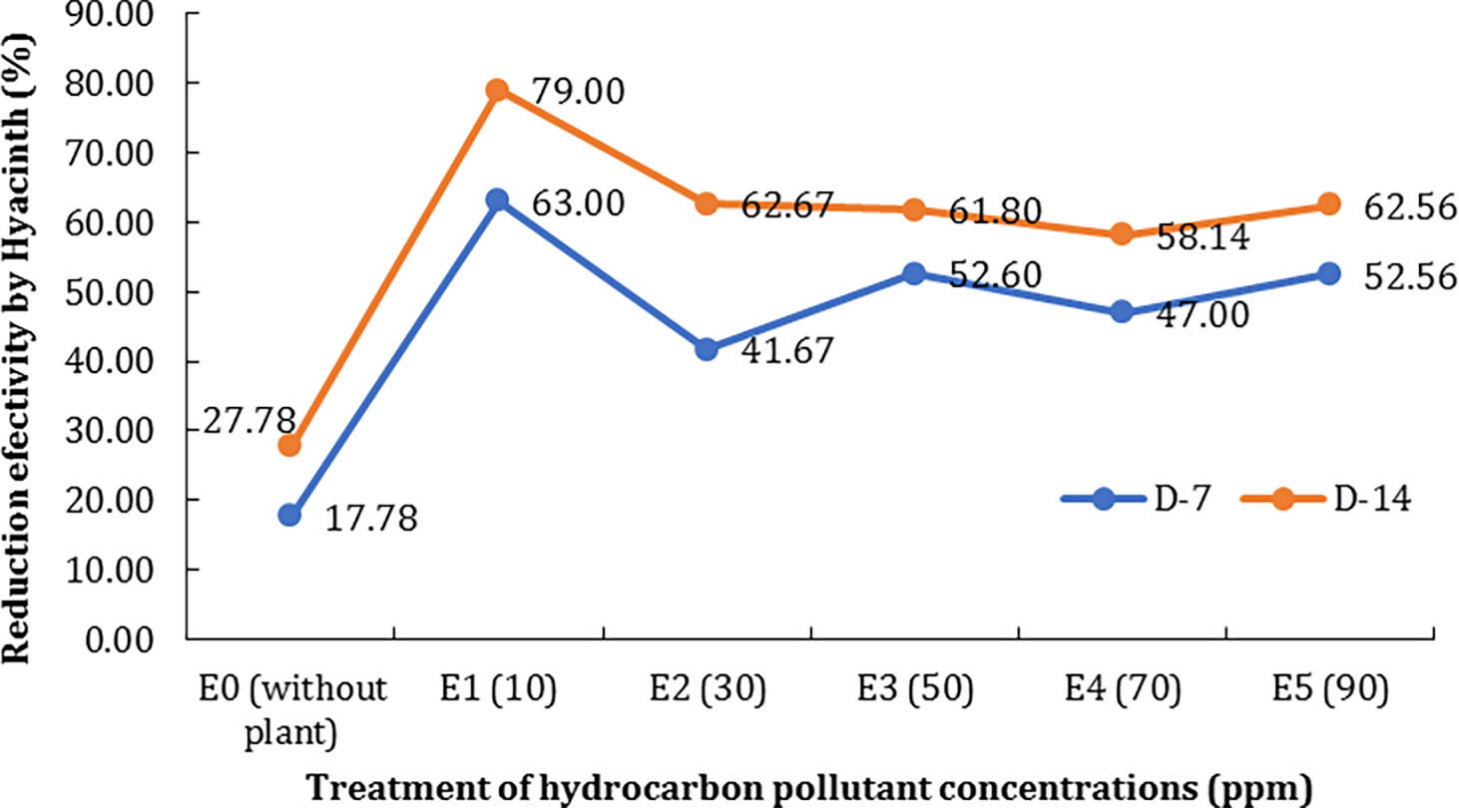
The effectiveness of the plant in decreasing total petroleum hydrocarbon (TPH).

The treatment without a phytoremediator (E0) can only reduce the TPH by as much as 17.78% after the 7 ^th^ day (D-7) and 27.78% after the 14 ^th^ day (D-14). In contrast, it ranged between 41.67% and 63% reduction using a phytoremediator. The highest effectivity of reductions was found at the treatment E10 (79%) with 10 ppm of petroleum hydrocarbon because the reduction process will be optimum at lower concentrations of pollutants ( Figure 1).

### Chlorophyll content and water quality

**Figure 2.**
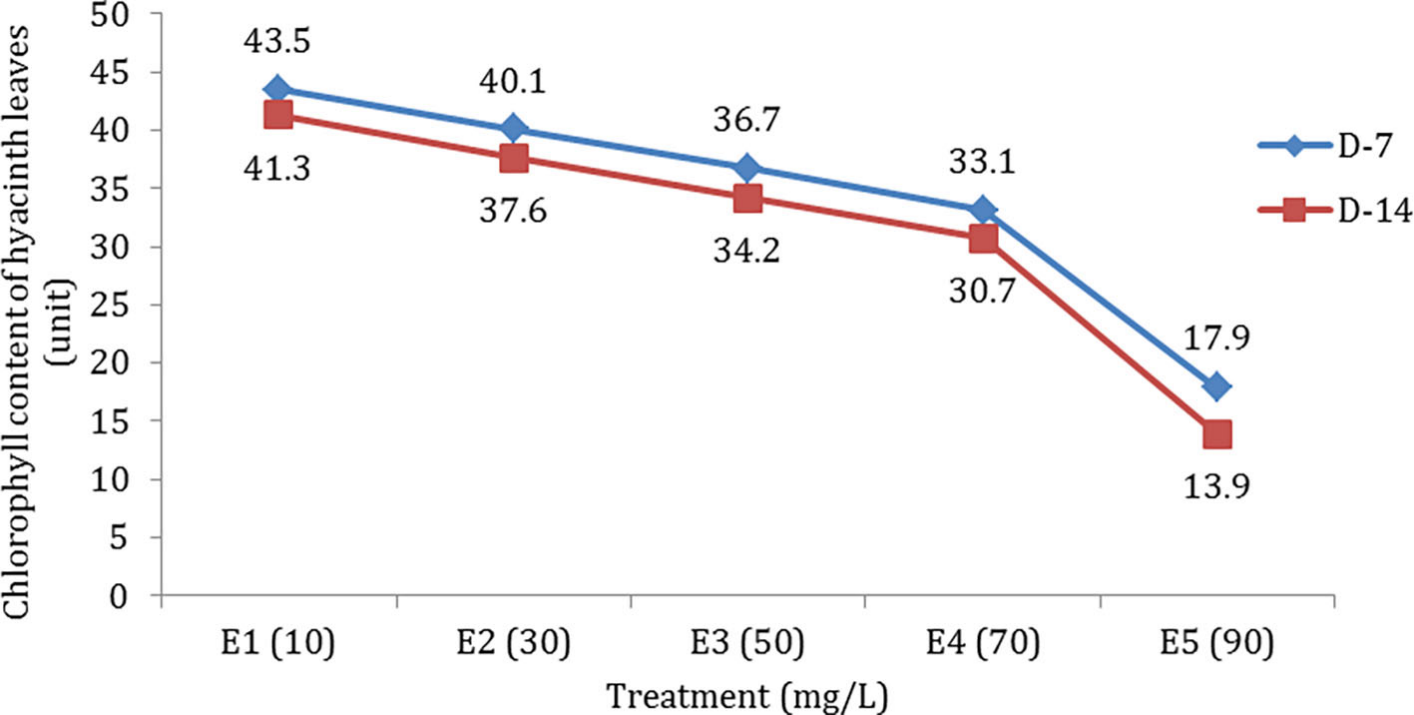
Chlorophyll content (mg/L) in hyacinth leaves based on observation time.

Chlorophyll is also an indication of the productivity of green plants and is one of the important elements in the process of photosynthesis. The results showed that the amount of chlorophyll in the leaves of the test plant (hyacinth) decreased in line with the increase in hydrocarbon pollutants and the length of observation time ( Figure 2). At the beginning of maintenance, the chlorophyll content in hyacinth leaves was 48.3 units. Along with TPH administration, there was a decrease in chlorophyll concentration in hyacinth leaves. In treating hydrocarbon pollutants (e.g. E5), the amount of chlorophyll encountered was 17.9 units on the seventh day and 13.9 on the 14 ^th^ day.

Differences in pollutant concentration treatment influence the chlorophyll content in hyacinth leaves (p<0.05). Another parameter of water quality is dissolved oxygen (DO). In control media (without plants), the average DO on the first day (D-1) was 3.0 mg/L; it increased to 3.3 mg/L and 3.5 mg/L after seven and 14 days consecutively. It indicated that the DO naturally increased with the pollutant hydrocarbon. However, the increases of DO were higher in the treatments with hyacinth phytoremediators; it was up to 20% compared to the treatments without the phytoremediator plants.

**Figure 3.**
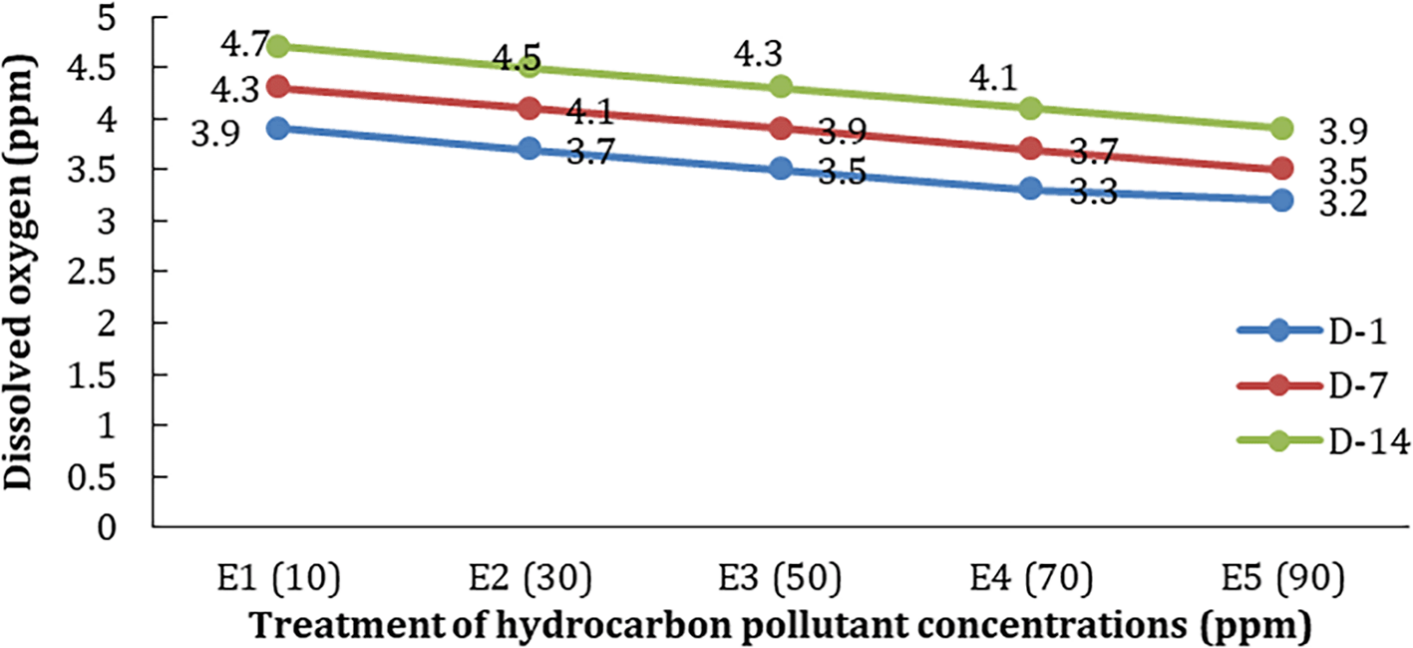
The decrease in dissolved oxygen (DO) in each treatment at the time of observation.

Variations in the concentration of hydrocarbon pollutants affect the dissolved oxygen of the test medium. The higher the concentration of hydrocarbon pollutants given, the higher the decrease in DO content in the test media water ( Figure 3).

However, the DO figures were slightly higher based on the days of observations and concentrations; D-14>D-7>D1. Temperature plays an important role in plant life and growth. A good temperature for the plant to grow is known as the optimum limit of temperature. At this limit, plants can grow well both in terms of morphology and physiology. Temperature conditions at the treatment with 10 mg/L hydrocarbon without phytoremediator’s plant (E0) were seen to decrease in line with the observation time ((D-1, D-2, and D-3); see Figure 3).

**Figure 4.**
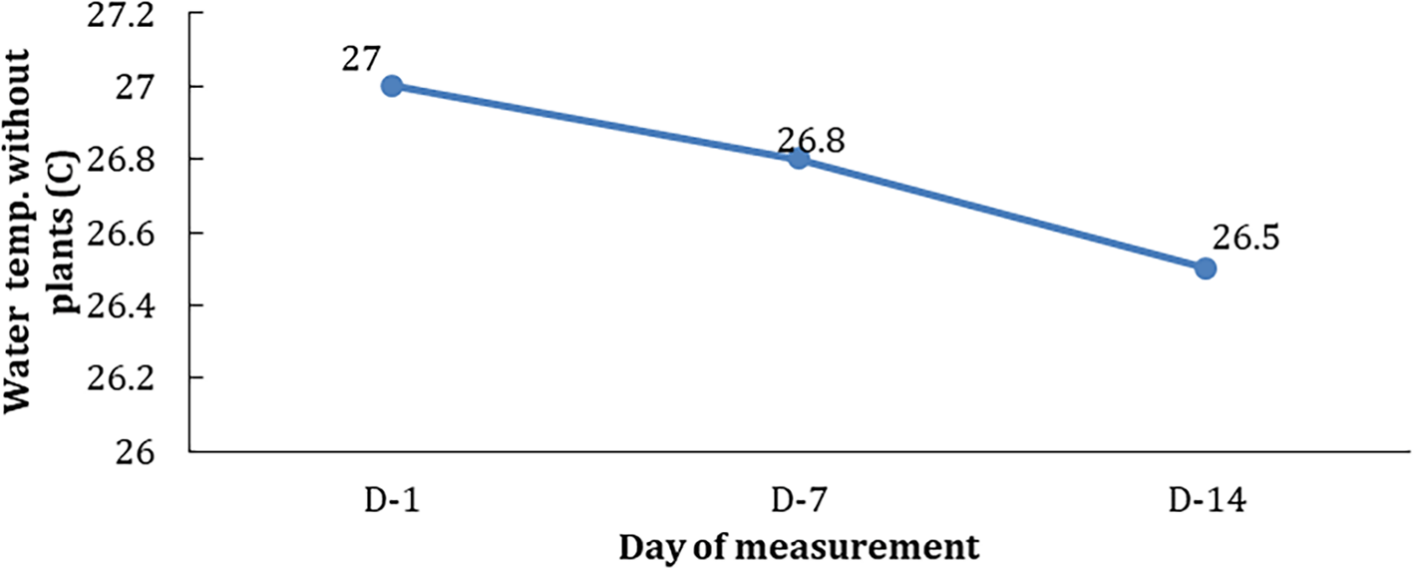
The temperature of the control medium (E0) on each day of observations.

Figure 4 shows that the presence of hydrocarbon pollutants affected the temperature of the water test medium. On the seventh and 14 ^th^ days, the same concentration of hydrocarbon pollutants occurs with a decrease in temperature by 0.2–0.3°C.

**Figure 5.**
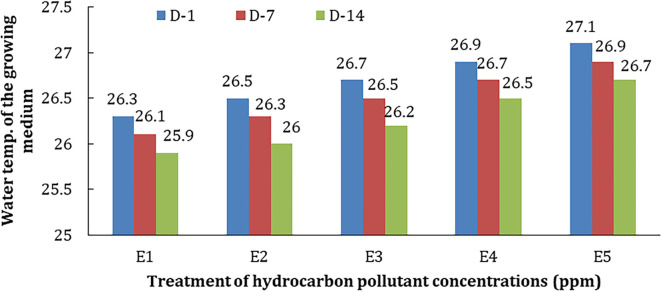
The temperature of treatment media at various concentrations during observation.

The temperature in the treatment medium with different concentrations of hydrocarbon pollutants and phytoremediators (E1–E5) can be seen in Figure 5.

Based on this figure, it can be seen that the temperature of the media increases in line with the increase in pollutant concentration and the length of time of observation. The increase in temperature may be closely related to the hydrocarbon reduction process that releases heat by phytoremediators when carrying out water purification. The increase in temperature may be closely related to the hydrocarbon reduction process that releases heat by phytoremediators when carrying out water purification and it might be due to the phytoremediator process and pollutant degradation.

**Figure 6.**
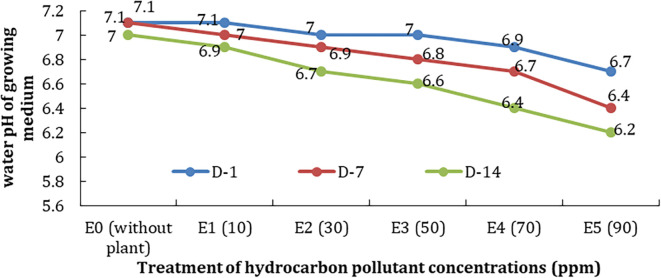
Decrease in water pH on the treatment of differences in concentration and observation time.

The degree of acidity (pH) is the concentration of hydrogen ions in the water. The pH of the growing water of treatments tended to decrease along with the higher concentration of pollutants; it might be due to the phytoremediator process and pollutant degradation. The decrease was 0.4–0.7 between D-1 and D-14 ( Figure 6).

## Discussion

Water hyacinth is a native freshwater plant from South America. ^21^^–^^23^ The results showed that this plant grows well at a salinity of 3 ppt; Ting *et al.*, 2018 ^24^ also reported that this plant can adapt and grow at a salinity of <5 ppt. In addition, this plant can act as a phytoremediator against toxic pollutants derived from petroleum hydrocarbons. The effectiveness of TPH reduction depends on the concentration and length of time of observation ( Table 1 and Figure 1). Meanwhile, optimum reduction efficiency is found in treatments with low concentrations (10 mg/L); i.e. 63% on D-7 and 79% on D-14. The process of reducing TPH by this plant is inseparable from its root system which acts as an absorbent and then spreads to all parts of the plant. ^25^^–^^27^ Some of the organic content contained in hydrocarbon pollutants is used as nutrients for plants; Oke *et al.*, 2020 ^28^ reported that the highest absorption of hydrocarbons by 100 g of hyacinth is 72%. The presence of biodegradable bacteria in the water hyacinth root system may also play a role in the process of reducing TPH in the test media, according to Xia & Ma (2006) ^29^ the presence of bacteria in roots can degrade pollutants by 12%. Thus, these plants can purify water contaminated by hydrocarbon pollutants.

Although water hyacinths can reduce TPH and purify water, it has an impact on the growth of the test plants. This condition was indicated by a decrease in the amount of chlorophyll in each treatment along with an increase in concentration and length of observation time ( Figure 2).

The decrease in the amount of chlorophyll on the leaves is inseparable from the disruption of metabolic processes in plants so that their productivity is disturbed. Plants exposed to pollutants for a certain time will experience chlorosis due to inhibition of the chlorophyll synthesis. ^30^ Of course, a decrease in the amount of chlorophyll affects the process of photosynthesis and enrichment of DO in waters.

In the control treatment without hyacinths (E0), it was seen that DO increased in line with the observation time ( Figure 3). The amount of DO in the treatment with hyacinths was higher than without plants or controls. The increase in DO may come from the diffusion of oxygen from the air as well as the photosynthetic activity of phytoplankton and test plants. Nevertheless, DO conditions decreased with increasing concentrations of hydrocarbons and the length of time of observation ( Figure 5). The same condition is also shown by the temperature and pH of the water test plant ( Figure 6). The decrease could be due to the reduction of chemical processes and the decomposition of TPH. Where in these processes, in addition to requiring oxygen, there is also absorption and release of heat, including the use of hydrogen ions which affect the pH of water.

## Conclusion

Hyacinth plants can reduce hydrocarbon pollutants. The effectiveness of the reduction of hydrocarbon pollutants by hyacinths can reach 79% at a hydrocarbon pollutant concentration of 10 mg/L. The presence of hydrocarbon pollutants has led to a decrease in chlorophyll in the leaves of hyacinths. Hyacinths can absorb organic matter without using oxygen for the decomposition process. Differences in pollutant concentration treatment influence the chlorophyll content in hyacinth leaves (p<0.05). Watered hydrocarbon pollutants are tolerated by hyacinth plants and can prevent water quality degradation.

## Data Availability

Zenodo: Reduction of Hydrocarbon Pollutants by Hyacinth Plants (Eichhornia crassipes), https://doi.org/10.5281/zenodo.7659979. ^31^ This project contains the following underlying data:
• EXCEL KONFILASI DATA ECENG GONDOK (1).xlsx (Effectiveness Hyacinth, water quality and Chlorophyll) Data are available under the terms of the Creative Commons Attribution 4.0 International license (CC-BY 4.0).
