# Supplementary material for: Reduction of hydrocarbon pollutants by hyacinth plants ( Eichhornia crassipes)
Source: F1000Res. 2023 Sep 12;12:728. Originally published 2023 Jun 22. [Version 2] doi: 10.12688/f1000research.131846.2 (PMC11310655; doi:10.12688/f1000research.131846.2)
Supplement: Peer review history

# Reviewer report: Reviewer response for version 2

**DOI:** 10.5256/f1000research.155785.r305684

**Published:** 2024-08-08

**Authors:** Santhana Krishnan

**Affiliations:** 1 Prince of Songkla University, Hat Yai, Songkhla, Thailand

The manuscript is not written well. It doesn’t follow the scientific standards.

 Some of the specific comments are provided below. 

 1. Abstract: In Methods section, TPH should be given in full form in the first appearance.

 2. I would see clusters of references for the first 3 lines. Authors must eliminate those multiple references. After that please check the manuscript thoroughly and eliminate ALL the lumps in the manuscript. This should be done by characterizing each reference individually. This can be done by mentioning 1 or 2 phrases per reference to show how it is different from the others and why it deserves mentioning.

 3. Introduction is very small with no information. For instance, in the introduction, you need to connect the state of the art to your paper goals. Please follow the literature review by a clear and concise state of the art analysis. This should clearly show the knowledge gaps identified and link them to your paper goals. Clearly discuss what the previous studies that you are referring to.

 4. In your discussion section, please link your empirical results with a broader and deeper literature review. For instance, RSM, Hyacinth significance in TPH removal, etc.

Is the work clearly and accurately presented and does it cite the current literature?

Partly

If applicable, is the statistical analysis and its interpretation appropriate?

Partly

Are all the source data underlying the results available to ensure full reproducibility?

Partly

Is the study design appropriate and is the work technically sound?

Partly

Are the conclusions drawn adequately supported by the results?

Partly

Are sufficient details of methods and analysis provided to allow replication by others?

Partly

Reviewer Expertise:

Environmental chemical Engineering

I confirm that I have read this submission and believe that I have an appropriate level of expertise to confirm that it is of an acceptable scientific standard, however I have significant reservations, as outlined above.

# Reviewer report: Reviewer response for version 2

**DOI:** 10.5256/f1000research.155785.r205638

**Published:** 2023-11-10

**Authors:** Anithadevi Kenday Sivaram

**Affiliations:** 1 The University of Newcastle, Callaghan, New South Wales, Australia

Thanks for revising the manuscript. However, some queries not addressed in version 2 need to be addressed.

**Specific comments:**

**Introduction:**

 The introduction section needs further improvement by addressing all the previous comments.

**Methods:**

1. Please include the initial measured concentration, their percentage recovery, and the basis for choosing different TPH concentrations for your study.
2. Please add a section on the TPH extraction and analysis procedure in water and plants and the QA/QC procedure.

** Results:**

1. Please include standard deviation (SD) in all the figures, wherever applicable.
2. Overall, the explanation of the results can be improved.

**Discussion:**

 The discussion part still focused on the results. Needs comprehensive discussion and comparison with other similar works.

**Conclusion:**  

 Please provide an overall conclusion rather than repeating the results in the section.

Is the work clearly and accurately presented and does it cite the current literature?

Yes

If applicable, is the statistical analysis and its interpretation appropriate?

Yes

Are all the source data underlying the results available to ensure full reproducibility?

Yes

Is the study design appropriate and is the work technically sound?

Partly

Are the conclusions drawn adequately supported by the results?

Partly

Are sufficient details of methods and analysis provided to allow replication by others?

Yes

Reviewer Expertise:

Phytoremediation, Ecotoxicology

I confirm that I have read this submission and believe that I have an appropriate level of expertise to confirm that it is of an acceptable scientific standard, however I have significant reservations, as outlined above.

# Reviewer report: Reviewer response for version 2

**DOI:** 10.5256/f1000research.155785.r205639

**Published:** 2023-10-03

**Authors:** Hemen Deka

**Affiliations:** 1 Ecology and Environmental Remediation Laboratory, Department of Botany, Gauhati University, Guwahati, Assam, India

The issues raised with the MS during the evaluation process have been addressed adequately. The MS has been significantly improved and can be accepted for publication.

Is the work clearly and accurately presented and does it cite the current literature?

Partly

If applicable, is the statistical analysis and its interpretation appropriate?

I cannot comment. A qualified statistician is required.

Are all the source data underlying the results available to ensure full reproducibility?

Yes

Is the study design appropriate and is the work technically sound?

Yes

Are the conclusions drawn adequately supported by the results?

Partly

Are sufficient details of methods and analysis provided to allow replication by others?

Partly

Reviewer Expertise:

Bioremediation and phytoremediation of hydrocarbons and heavy metals

I confirm that I have read this submission and believe that I have an appropriate level of expertise to confirm that it is of an acceptable scientific standard.

# Reviewer report: Reviewer response for version 1

**DOI:** 10.5256/f1000research.144729.r196910

**Published:** 2023-08-29

**Authors:** Hemen Deka

**Affiliations:** 1 Ecology and Environmental Remediation Laboratory, Department of Botany, Gauhati University, Guwahati, Assam, India

**Overall comments:**

 The authors of the manuscript conducted research on “Reduction of hydrocarbon pollutants by hyacinth plants ( *Eichhornia crassipes*)’—however, the authors must make it more evident in their paper to outline the design of the study and methods. The manuscript's writing and discussion are not up to mark, requiring thorough improvements across all sections. In addition to addressing scientific shortcomings, the entire paper necessitates careful editing and enhancement. Make sure that your manuscript has been thoroughly edited for proper use of the English language and accurate technical terminology.

 Authors should have a strong justification for choosing only hyacinth plants ( *Eichhornia crassipes*). Why not other aquatic plants? It should be mentioned more elaborately in the introduction part itself.

 Certain errors in spelling are also noticeable. Please correct these mistakes.

**Specific comments:**

**Abstract:**

- Requires modifications. Please incorporate more numerical date into the abstract.

**Introduction**:

- Introduction part is very poor. It should be expanded.
- This section lacks clear cut objective of the study. Also highlight the significance of the topic, identify research gaps, and explain how this paper will contribute theoretically, empirically, and methodologically.

**Research methods:**

- How was petroleum hydrocarbon was added to the water? Isn’t it non-polar? Write the detail procedure.

***Petroleum hydrocarbon observations***

- What is the concentration of total petroleum hydrocarbons in control without plants?
- How do you calculate total petroleum hydrocarbons, pH, DO etc.? Give standard protocol.
- Table 1: concentration of petroleum hydrocarbon in E0=?

***Data analysis***

- In which significance level data were analyzed?

**Result: **

- Please rewrite the section so that it becomes readable for readers.

***Total petroleum hydrocarbon concentration***

- "This is thought to be due to evaporation (E0)" - you should mention this line in ‘Discussion’.
- 3rd para: What is E10?
- Table 2, 2nd column-last row: Is Con. of E0=90? I can’t understand. Why do you keep only one concentration for total petroleum hydrocarbons in E0? Why not other concentrations like 10, 30, 50 and 70 ppm as mentioned in the treatments with phytoremediator?
- The figures and tables can't communicate on their own. Instead, the authors should explain the insights revealed by the findings. In Figure 2, 5 and 6, check the Y-axis title and give proper units
- You have taken three replicas, right? Then why didn’t you mention about standard deviations in any of the figures. Also, In each figure, instead of putting values above the line or bar, you should give a clear cut idea about significant variations in the values.

***Chlorophyll content and water quality***

- In the treatment without hydrocarbons (E0): What is E0? Previously, in Table 2 you mentioned E0 as without phytoremediator? Here you have mentioned E0 as without hydrocarbons. Please give a detail information about the treatments with proper notation.
- "Temperature conditions at the treatment with 10 ppm hydrocarbon without phytoremediator’s plant (E0) were seen to decrease in line with the observation time ((D-1, D-2, and D-3)" - Isn’t it D-1, D-7, D14?
- In Figure 3 red line indicates ‘with plant’- What is the notation for this treatment? Please give in details.
- "Based on this figure, it can be seen that the temperature of the media increases in line with the increase in pollutant concentration and the length of time of observation." - The figure is not indicating itself the findings.
- "The increase in temperature may be closely related to the hydrocarbon reduction process that releases heat by phytoremediators when carrying out water purification" & "ii) it might be due to the phytoremediator process and pollutant degradation." - these two sentences should be written in discussion part.
- "The pH of the growing water of treatments..." - What is 'growing water'?
- I recommend for revising the discussion to better address the theoretical, empirical, policy, and methodological issues presented in the paper. Also, more recent literature in the introduction and discussion section is of required. For better representation your MS, you may add new references and work; the following are suggested references: Chakravarty & Deka (2021) ^1^; Chakravarty *et al.* (2022) ^2^; Kalita *et al.* (2022) ^3^; Boruah *et al. *(2020) ^4^; Chakravarty *et al. *(2023) ^5^.

**Conclusion: **

- The concluding section is quite brief. Kindly present the specifics of your work concisely, making it easily accessible to everyone.

**References:**

- All references according to the journal format.

Is the work clearly and accurately presented and does it cite the current literature?

Partly

If applicable, is the statistical analysis and its interpretation appropriate?

I cannot comment. A qualified statistician is required.

Are all the source data underlying the results available to ensure full reproducibility?

Yes

Is the study design appropriate and is the work technically sound?

Yes

Are the conclusions drawn adequately supported by the results?

Partly

Are sufficient details of methods and analysis provided to allow replication by others?

Partly

Reviewer Expertise:

Bioremediation and phytoremediation of hydrocarbons and heavy metals

I confirm that I have read this submission and believe that I have an appropriate level of expertise to confirm that it is of an acceptable scientific standard, however I have significant reservations, as outlined above.

## References

[1] : Enzymatic defense of Cyperus brevifolius in hydrocarbons stress environment and changes in soil properties. *Sci Rep* .2021;11(1) : 10.1038/s41598-020-80854-5 718 10.1038/s41598-020-80854-5 33436992 PMC7804112

[2] : Ecological risk assessment of priority PAHs pollutants in crude oil contaminated soil and its impacts on soil biological properties. *J Hazard Mater* .2022;437: 10.1016/j.jhazmat.2022.129325 129325 10.1016/j.jhazmat.2022.129325 35716561

[3] : Understanding biochemical defense and phytoremediation potential of Leucas aspera in crude oil polluted soil. *Environ Sci Pollut Res Int* .2022;29(38) : 10.1007/s11356-022-19922-4 57579-57590 10.1007/s11356-022-19922-4 35355178

[4] : Phytosociology and antioxidant profile study for selecting potent herbs for phytoremediation of crude oil-contaminated soils. *Environ Monit Assess* .2020;192(12) : 10.1007/s10661-020-08721-4 766 10.1007/s10661-020-08721-4 33210208

[5] : Anthracene removal potential of green synthesized titanium dioxide nanoparticles (TiO2-NPs) and Alcaligenes faecalis HP8 from contaminated soil. *Chemosphere* .2023;321: 10.1016/j.chemosphere.2023.138102 138102 10.1016/j.chemosphere.2023.138102 36764617

# Reviewer report: Reviewer response for version 1

**DOI:** 10.5256/f1000research.144729.r192190

**Published:** 2023-08-21

**Authors:** Anithadevi Kenday Sivaram

**Affiliations:** 1 The University of Newcastle, Callaghan, New South Wales, Australia

In this manuscript, the author applied phytoremediation using water hyacinth to remove the total petroleum hydrocarbons from the water. The work is presented with current literature. However, there are issues with the experimental techniques and methods. Firstly, the method section lacks information on how the TPH concentration in water is measured after spiking, the QA/QC procedure for TPH analysis and spiking recovery (%). Secondly, the procedure for other parameters (DO, Salinity) were missing. Finally, it is important to include bioaccumulation in the hyacinth by measuring the concentration of TPH in the hyacinth.

**Specific comments:**

**Abstract:**

1. Please rewrite the last three sentences in the background.
2. Please give the details on the treatments and control clearly for better understanding. As per the results, there are 2 controls i) one without plant grown in 90 ppm TPH and ii) another with plant and without TPH concentration for biochemical and TPH analysis.
3. It is good to change the concentration expression to mg/L rather than expressing it as ppm. Also, measured TPH concentration can be provided rather than the nominal concentration.

**Introduction:** Too short. It would be better to add about the i) toxic properties of TPH; ii) remediation methods followed; iii) Significance of phytoremediation; iv) why water hyacinth and previous reports to support your plant and TPH selection.

**Materials and Methods:**

1. Please mention the controls used for the experiment.
2. It is recommended to include the measured concentrations and the basis for choosing different TPH concentrations.
3. Please add a section on the TPH analysis procedure in water and plants and QA/QC procedure.
4. Please add on the pH, DO and salinity experiment procedure.

**Result:**

1. Table 2. It is better to provide the results in terms of removal percentage by merging the results in Fig.1 to Table 2 for better clarity.
2. Fig.2 can be removed.
3. It is suggested to include standard deviation (SD) in all the figures.
4. Figure 7. Is the E0 with TPH conc? 
5. Overall, the explanation of the results can be improved.

**Discussion: **The discussion part focused more on results. Needs comprehensive discussion.

**Conclusion:** Explains only results and also repeats the contents present in the abstract and result section. Please revise.

Is the work clearly and accurately presented and does it cite the current literature?

Yes

If applicable, is the statistical analysis and its interpretation appropriate?

Yes

Are all the source data underlying the results available to ensure full reproducibility?

Yes

Is the study design appropriate and is the work technically sound?

Partly

Are the conclusions drawn adequately supported by the results?

Partly

Are sufficient details of methods and analysis provided to allow replication by others?

Yes

Reviewer Expertise:

Phytoremediation, Ecotoxicology

I confirm that I have read this submission and believe that I have an appropriate level of expertise to confirm that it is of an acceptable scientific standard, however I have significant reservations, as outlined above.
